# Neonatal thyrotropin levels and auditory neural maturation in full-term newborns

**DOI:** 10.1371/journal.pone.0253229

**Published:** 2021-06-16

**Authors:** Leticia Valerio Pallone, Laura Carvalho Navarra, Gleice Aline Gonçalves, Felipe Alves de Jesus, Debora Gusmão Melo, Rodrigo Alves Ferreira, Carla Maria Ramos Germano

**Affiliations:** Department of Medicine, Federal University of São Carlos, São Paulo, Brazil; Universidad de Chile, CHILE

## Abstract

**Objective:**

This study aimed to look for a possible relationship between thyrotropin (TSH) values from neonatal bloodspot screening testing and newborn lower auditory pathway myelinization evaluated using the brainstem evoked response audiometry (ABR) test.

**Methods:**

Sixty-two healthy full-term newborns without perinatal problems were enrolled in the study. TSH results were collected from neonatal bloodspot screening data and were below the test cut-off level (15μUI/mL). The TSH test was performed between three and seven days, and the ABR test was performed in the first 28 days of life. The newborns were divided into two groups: Group 1 (n = 35), TSH between 0 and 5μUI/mL, and group 2 (n = 27), TSH between 5 and 15μUI/mL. Data are presented as mean ± SD, median, or percentage, depending on the variable.

**Results:**

Wave latency and interpeak interval values for Groups 1 and 2 were as follows: Wave I: 1.8 ± 0.1 and 1.7 ± 0.1; Wave III: 4.4 ± 0.1 and 4.4 ± 0.1; Wave V: 6.9 ± 0.1 and 6.9 ± 0.1; interval I–III: 2.6 ± 0.1 and 2.6 ± 0.1; interval I–V: 5.1 ± 0.1 and 5.1 ± 0.1; interval III–V: 2.4 ± 0.1 and 2.4 ± 0.1. There were no significant differences in ABR parameters between groups 1 and 2 (p > 0.05). Multiple regression analysis showed a slight significant negative correlation between TSH and wave I values (standardized β = −0.267; p = 0.036), without observing any relationship with the other ABR waves recorded.

**Conclusions:**

This study investigated the relationship of TSH and auditory myelinization evaluated by ABR. It did not show a significant change in lower auditory pathway myelinization according to TSH levels in newborns with TSH screening levels lower than 15 μUI/mL.

## Introduction

Thyroid hormone (TH) is involved in a wide range of physiological processes [1]. In the development of the central nervous system (CNS) during fetal life and throughout the first years of life, vascularization, myelinization, dendritic arborization, synapse formation, neuronal migration, cell differentiation, and gene expression processes depend on the presence of TH [2, 3]. Adequate levels of TH during this period are essential [3], and perinatal deficiency of these hormones may result in brain maturation disturbance, intellectual deficit, and, in some cases, impairment of language, memory, and vision. These disorders can occur within different spectra and forms of involvement according to the period in which hypothyroxinemia occurs [4].

The actions of TH are fundamental in the development of the auditory system [5]. Richter et al. (2011) demonstrated that TH is fundamental for the morphological and functional development of the cochlea [6]. Classical studies have already demonstrated a significant association between congenital hypothyroidism (CH) and hearing impairment, well above the prevalence in the general population [5]. The presence of significant hearing disturbances in individuals with CH highlights the influence of TH on the embryonic development of the auditory system, and the critical period of maturation of the TH-dependent auditory system seems to be in the first and second trimesters of pregnancy [7].

In a study aimed at identifying speech-language manifestations in children with CH, disorders such as delayed oral language onset, speech exchange, unintelligible speech, and hearing impairment were found. There was a relationship between the presence of complaints, time of diagnosis, and the beginning of CH treatment [8]. However, research evaluating other hearing-dependent skills, such as communication disorders or electrophysiological studies of the auditory system, are scarce [9, 10].

The auditory brainstem response test (ABR) records the electrophysiological activity of the auditory system from the cochlear nerve to the brainstem level, assessing the cochlear nuclei and superior olivary complex, and bridging to the mesencephalic inferior colliculus [11]. ABR tracing can be analyzed using both qualitative and quantitative parameters. The analysis is based on the latency/intensity and amplitude/intensity curve of the recorded waves and intervals carried out at different levels of stimulation (near threshold or supra-threshold. For that reason, one way to analyze ABR data is assessing the absolute wave latency (I-wave, III-wave, and V-wave) and interpeak intervals (I–III, III–V, and I–V) [11]. Wave latency time and intervals between wave peaks correlate with nerve conduction velocity and are influenced by the degree of myelinization of the auditory pathway. Therefore, these values constitute an indirect measure of neural myelination at different levels of the auditory pathway. The relationship between these latencies and myelination is inversely proportional; hence, lower latency represents higher nerve conduction velocity and better myelination [12].

The ABR test has been used as a method to evaluate the auditory system in individuals with CH, and changes have been found in infants, adolescents, and adults with this disease [9]. However, there are no data in the literature on auditory system analysis in newborns with clinical and subclinical CH.

Since 2015, the Brazilian Ministry of Health has adopted a thyroid-stimulating hormone (TSH) value above 15 μIU/mL per immunometric assay as the cut-off value for newborn recall [13]. There is a consensus that RN with increased TSH values (TSH ≥ 15 mIU/mL) must be investigated according to the screening program flowchart. However, this value is higher than that adopted by neonatal screening programs in other countries and within some countries’ states [14]. In 2018, Kilberg et al. suggested that US neonatal screening programs were missing newborns with mild and persistent TSH elevations, who could have CH and its health consequences, due to the lack of adequate standardization of TSH values [15]. Christensen-Adad et al. (2017) also demonstrated that 9% of children with TSH values between and 5–10 μIU/mL (screening test cut-off = 10 μIU/mL), who did not normalize these values over time, had CH that would not have been diagnosed by the national recommended cut-off value [16].

Although the scientific literature recognizes that there is a need to establish an optimal TSH level for use in neonatal screening to diagnose children with CH who deserve treatment, in order to maximize benefits, there is no consensus on the ideal TSH value to fulfill this task [17]. Therefore, the aim of the present study was to conduct a study that looked for a possible relationship between the early auditory evoked response and TSH levels in healthy newborns, considering the fundamental role of thyroid hormones in the development of the auditory system.

## Methods

This cross-sectional, quantitative study was conducted in Sao Carlos (SP, Brazil) from August 2016 to January 2018 at a maternity hospital responsible for the delivery of all births during this period. This research was approved by the Sao Carlos Federal University Human Research Ethics Committee (CAAE 35584114.2.0000.5504) and participation was authorized by signing the Informed Consent Declaration. Those considered eligible for the study were healthy newborns of both sexes with more than 37 weeks of gestational age, born in the maternity hospital during the study, and whose parents or legal representatives signed the free and informed consent form.

One hundred and sixty-five (165) newborns, out of 235 eligible ones, agreed to participate in all the stages of the study. A registration form, validated in a previous study from the same group, was completed with maternal and newborn perinatal information obtained from medical records and interviews with mothers [18]. All newborns underwent hearing screening using the otoacoustic emission test (OAE) and those who passed in this screening underwent audiological evaluation based on the ABR test. The 165 mothers also answered the following questionnaires: T-ACE, an alcohol-screening validated questionnaire adapted to identify risk drinking during pregnancy, and the Fagerström Test, a standard validated instrument for assessing the level of addiction to nicotine.

Regarding the exclusion criteria (after direct questioning and medical record research), we excluded newborns with risk indicators for hearing loss, such as family history of congenital hearing impairment in first-degree relatives; congenital infections (toxoplasmosis, rubella, cytomegalovirus, herpes, syphilis, HIV), weight ≤ 2500 g, genetic syndromes, presence of craniofacial abnormalities, perinatal complications (1- or 5-minute Apgar score less than 7), hemolytic disease, hypoglycemia, prolonged jaundice, neonatal sepsis or intensive care unit (ICU) permanence, and newborns with congenital anemia (hematocrit < 42%) or some degree of iron deficiency (ferritin ≤ 75 mg/mL). Newborns whose mothers reported the use of alcohol (T-ACE score equal to or greater than 2) or any illicit drugs (excluded by a direct question: drug use, yes or no), and a significant nicotine dependence (Fagerström test score equal to or greater than 5 during pregnancy) were also excluded because any of these comorbidities alone could change the ABR results. Newborns of mothers who used levothyroxine during pregnancy or had a thyroid disease diagnosis registered in their medical records, and newborns with increased TSH values in the neonatal screening test (blood spot TSH ≥15 mUI /mL) were also excluded. After applying the exclusion criteria, 62 newborns were included in the analysis of neonatal ABR and TSH results (S1 Fig).

The bloodspot TSH, determined by an immunometric assay according to the guidelines of the National Screening Program [13], was carried out between postnatal days 3 and 7, and the results were obtained from the Epidemiological Surveillance office. The cut-off value adopted was 15 μUI/mL.

The hematocrit was measured after centrifugation in an appropriate tube under standardized conditions, using the method proposed by Wintrobe– 1987. Ferritin determination was performed using a solid-phase chemiluminescent immunometric enzyme assay (IMMULITE/IMMULITE 1000 FERRITIN—Siemens Healthcare Diagnostics UK). Samples were processed in a certified laboratory within 4 h of collection.

All audiological evaluations (OAE and ABR) were performed, and their results were analyzed by the same qualified phonoaudiologist professional, who was not previously informed of the TSH results. The OAE was performed with a “pass or fail” screening mode in newborns within 48 hours of life. ABR was performed in the diagnostic mode between 2 and 28 days of postnatal age. Sedation was not required, and the examination was carried out with the newborn sleeping in a quiet environment with electrical insulation and dimmed light. The equipment for performing the ABR was a BIO-LOGIC NAVIGATOR PRO^®^ Equipment (Stimulus: “click” type; Polarity: alternating). Headphones with monaural stimulation and sound stimulus with an intensity of 80 dBNA were used (HOOD STANDARDIZATION—1998). The impedance between the electrodes was considered to be less than 3 kΩ, according to the instructions of the equipment. The parameters utilized were: alternating polarity clicks at a rate of 27.7 clicks/s; low pass filter: 100 Hz; high pass filter: 1500 Hz; total stimuli: 2000; window analysis: 10.66 ms. Each record was duplicated to ensure reproducibility of the results. The degree of myelination was graded according to the latency and interpeak intervals of waves I, III, and V, considering the best value between both ears of each newborn, that is, the one with the shortest conduction time.

The 62 newborns that comprised the final sample were divided into two groups according to their TSH values: Group 1, TSH ≤ 5 μUI /mL; and Group 2, TSH > 5 and < 15 μUI /mL.

The formula $n=2{\left(Z_{1-\frac{\alpha }{2}}+Z_{1-ß}\right)}^{2}/{ES}^{2}$ was used to calculate the sample size necessary to find a clinically meaningful difference in Wave I values with 95% confidence and a probability of 10% (power 90%) for the occurrence of a Type II error (β risk), resulting in a sample size equal to 21 for each group (total sample = 42). For Effect size (ES) determination (ES = (μ1-μ2)/σ), the relevant mean difference for ABR waves (μ1-μ2) was established as 0.1, and σ was 0.1, based on clinically relevant differences in ABR waves described in the literature [18]). Data are presented as mean ± SD, median (minimum-maximum), or percentage, depending on the variable. The assumption of data normality (Gaussian distribution) was verified by the Shapiro-Wilk test, and the statistical significance of the differences was determined by the parametric t-test or the non-parametric t-test (Mann–Whitney) depending on the results. Fisher’s exact test was used to calculate differences in frequency. Pearson or Spearman binary correlation tests were used to investigate the association between the variables, depending on their distribution. Multiple regression analysis was performed with TSH as the dependent variable and ABR waves and intervals as the independent variables using the stepwise method. In this method, non-contributing variables were excluded by a step-by-step statistical process to identify the most significant correlations. In order to verify the quality of the adjusted model, the R^2^ (coefficient of determination) was calculated and the significance of the model was stablished by ANOVA. We presented the complete multiple regression analysis data in S1 File to reinforce its adequacy (S1 File). Statistical analyses were performed using SPSS version 24 (SPSS, Chicago, IL, USA), and a p-value < 0.05 was considered significant.

## Results

Among the 62 full-term newborns who underwent ABR, 35 had TSH less than or equal to 5 μIU/mL, and were included in Group 1; 27 had TSH test results greater than 5 μUI/mL and less than 15 μUI/mL, and were included in Group 2.

There were no significant differences between the groups in terms of maternal age, type of health care (public health care or supplementary health care), number of pregnancies, previous miscarriages, number of prenatal consultations, systemic arterial hypertension during pregnancy, or type of delivery. There was also no difference between the characteristics of newborns: sex, weight, length, type of health care, 1- and 5- minute Apgar scores, cord blood hematocrit and ferritin levels, gestational age at birth, and postnatal age when performing the ABR test (Table 1).

**Table 1:** Characteristics of the study population according to TSH levels.

Perinatal Data		Group 1	Group 2	p value
		TSH ≤ 5	5 > TSH < 15	
**Maternal age**				
Mean ± SD [median]		31 ± 6.5 [31]	31 ± 5.2 [31]	0.986^a^
**Health care**	**Supplemental** n (%)	20 (57.0)	13 (48.0)	0.798^c^
	**Public** n (%)	15 (43.0)	14 (52.0)	
**Number of prenatal visits** median (min-max)		10 (7–14)	10 (4–15)	0.929^a^
**Maternal hypertension** n (%)		2 (6.0)	3 (11.0)	0.650^c^
**RN sex**	**Female** n (%)	17 (49.0)	11 (41.0)	0.612^c^
	**Male** n (%)	18 (51.0)	5 (59.0)	
**Gestational age (days)**				
Mean ± SD [median]		274.0 ± 5.3 [273]	274.9 ± 7.1 [275]	0.721^b^
**Postnatal age (days)**				
Mean ± SD [median]		14.6 ± 6,2 [15]	15.0 ± 5.6 [14]	0.797^a^
**Birth weight (g)**				
Mean ± SD [median]		3312 ± 331.9 [3315]	3275 ± 370.8 [3220]	0.483^b^
**Birth length (cm)**				
Mean ± SD [median]		48.4 ± 1.9 [48]	48.1 ± 3.5 [48.5]	0.818^a^
**Ferritin (ng/ml)**,				
Mean ± SD [median]		152.6 ± 92.7 [132]	159.3 ± 76.5 [152,4]	0.594^a^
**Hematocrit (%)**				
Mean ± SD [median]		47.6 ± 3.8 [47.5]	47.0 ± 3.6 [46,6]	0.427^a^
**TSH (μUI/L)**				
Mean ± SD [median]		3.3 ± 1.0 [3,3]	7.5 ± 2.3 [6,6]	**< 0.0001**^a^^,^^d^

^a^ Mann-Whitney test;

^b^ t-test;

^c^ Fisher’s exact test.

^d^ p < 0.05.

Waves I, III, and V latencies and the interpeak intervals I–III, I–V, and III–V for groups 1 and 2, as well as the significance of the test comparing each variable between the groups (t-test or Mann–Whitney test according to the results of the Shapiro-Wilk test), are shown in Table 2. There were no significant differences in ABR waves I, III, and V or intervals I–III, II–V, and I–V between the two groups.

**Table 2:** ABR waves latencies and intervals in groups 1 and 2.

	Group 1	Group 2	*p* value
	TSH ≤ 5 μUI/mL (n = 35)	5 > TSH < 15 μUI/mL (n = 27)	
**Wave I (ms)** (mean ± SD)	1.8 ± 0.1	1.7 ± 0.1	0.143^b^
**Wave III (ms)** (mean ± SD)	4.4 ± 0.1	4.4± 0.1	0.169^a^
**Wave V (ms)** (mean ± SD)	6.9 ± 0.1	6.9 ± 0.1	0.435^b^
**Interval I-III (ms)** (mean ± SD)	2.6 ± 0.1	2.6 ± 0.1	0.823^a^
**Interval I-V (ms)** (mean ± SD)	5.1 ± 0.1	5.1 ± 0.1	0.864^b^
**Interval III-V (ms)** (mean ± SD)	2.4 ± 0.1	2.4 ± 0.1	0.372^b^

^a^ Mann-Whitney test;

^b^t-test

**Fig 1:**
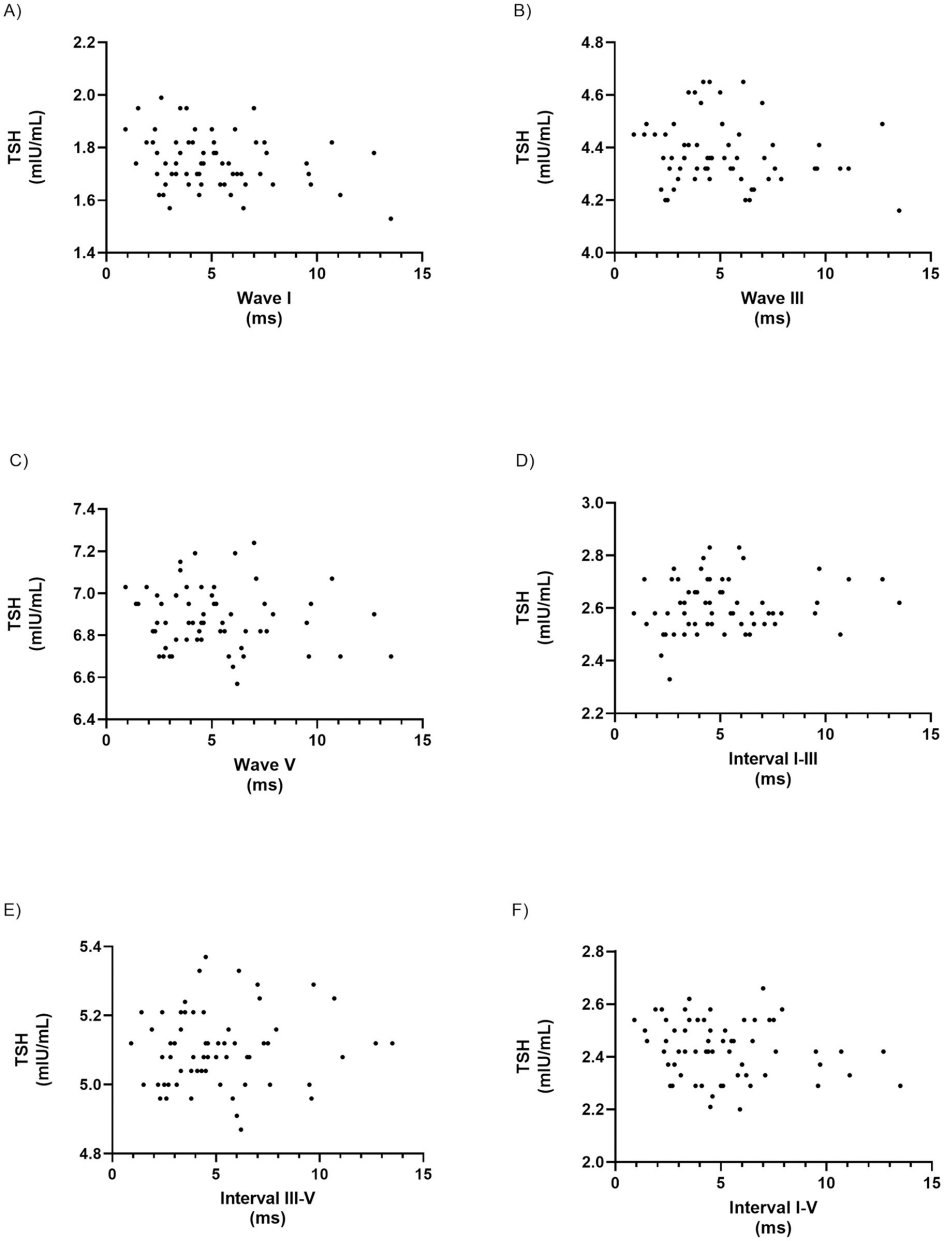
Scatter plot graphs. TSH levels (μUI/mL) vs wave I (A), wave III (B), wave V (C), and intervals I–III (D), III–V (E), I–V (F) latencies in milliseconds (ms).

The scatter plot graphs of TSH levels vs. wave and interval latencies are depicted below (Fig 1).

The results of Spearman’s correlation analysis between TSH and each ABR variable are shown in Table 3. There was no significant nonparametric correlation between TSH levels and ABR waves and interval.

**Table 3:** Spearman’s correlation analysis between TSH and each ABR wave and interval.

	TSH (μUI/mL)
**Wave I (ms)** r (p)	-0.226 (0.077)
**Wave III (ms)** r (p)	-0.163 (0.206)
**Wave V (ms)** r (p)	-0.108 (0.402)
**Interval I-III (ms)** r (p)	0.144 (0.264)
**Interval I-V (ms)** r (p)	0.090 (0.488)
**Interval III-V (ms)** r (p)	-0.202 (0.115)

r = correlation coefficient; p = significance.

To further investigate the association of TSH with waves and interval latencies, we performed a multiple regression analysis with TSH as the dependent variable, and waves (I, III, and V) and intervals (I-III, III-V, and I–V) as the independent variables (Table 4) (S1 File).

**Table 4:** Multiple regression analysis of TSH (dependent variable) with wave I, wave III, wave V, intervals I-III, III-V and I-V (independent variables) in a stepwise model.

Model 1	Unstandardized coefficient B	Standardized coefficient β	p	R	R square (R^2^)	Adjusted R square (R^2^)	^Std. Error of the estimate^	Durbin-Watson
**Constant**	17.491		0.004*					
**Wave I**	-7.072	-0.267	0.036*	.267^a^*	.071	.056	2.635	1.774

Model summary.

^a^Predictors: Constant, wave I. Dependent variable: TSH; Variables removed by the program: wave III, wave V, intervals I-III, III-V and I-V; ANOVA = 0.036;

* p<0.05.

The analysis showed a significant negative correlation between TSH and wave I.

## Discussion

CH poses a risk for hearing impairment, and Bruno et al. (2015) showed that 25% of children with CH detected by neonatal screening and treated properly had a mild and subclinical degree of hearing impairment in adolescence [7]. This suggests that auditory pathway changes may be already present at birth. Another study conducted with 112 CH patients older than five years of age demonstrated more frequent changes in cognitive functions, such as auditory attention, figure-background ability, and auditory memory, all related to central auditory processing [19].

Whole blood TSH levels from neonatal bloodspot proved to be adequate as a screening method for the detection of CH and correlated with serum TSH levels [20]. However, there are variations in the cut-off value adopted by neonatal screening programs in different countries, and even within countries, which implies a non-uniformity in the rate of retesting and pathology prevalence statistics. Therefore, in some states, the cut-off values recommended by the local screening programs were between 4.5 and 10 μIU/mL [14], well below the values recommended by the National Screening Program. Silvestrin et al. (2017) suggested that a cut-off value of 5.03 μIU/mL could result in a higher sensitivity (100%) and specificity (93.7%) for the detection of CH [14]. Another study showed that a percentage of newborns with TSH values below 10 and hyperresponsiveness to the thyrotropin-releasing hormone (TRH) test was diagnosed with CH during follow-up [21]. A recent study conducted in the United Kingdom, where the TSH cut-off value was 10 μIU/mL, demonstrated that 33% of newborns with TSH values between and 8–10 μIU/mL had CH, which was later confirmed [22].

Although the evaluation of TSH levels is an appropriate strategy for detecting primary CH, as it has high sensitivity and specificity, the cut-off value of the neonatal bloodspot screening test must be optimized. The aim is to minimize the false positive rate while maintaining a low percentage of false negatives since the recall from newborn screening test determines the need for clinical reevaluation and follow-up, and is a source of stress for parents [23]. The cut-off TSH value also needs to be continually reviewed, as recent data in the literature show that even children diagnosed early with CH, who started treatment at the recommended time, experienced changes in neurocognitive development during the clinical follow-up [24, 25], suggesting that these changes were established earlier in life.

After conducting a literature review in national and international databases, we found no recent articles that have analyzed the results of ABR in newborns according to the TSH values from the neonatal screening test, especially below the cut-off level of 15 μUI/mL.

In this study, multiple regression analysis showed a statistically significant association between wave I and TSH levels in the multivariate model with a low standardized R value (r = -0.26; p = 0.036). Wave I assess the action potential of the lower part of the auditory pathway [26], and this finding suggests that this could be the area of the auditory system most sensitive to thyroid hormone actions during the perinatal period. This result needs to be confirmed and its relevance more thoroughly investigated in further studies with a higher sample to improve the analysis precision.

Considering a cut-off of 5 μUI/mL, we did not find a significant difference in ABR data, that is, in the myelination of the auditory pathway between the groups with TSH higher or below this value. This is relevant, as newborns in the present study had TSH values below the cut-off value of the neonatal screening test (15 μUI/mL) and would not be included in a protocol for further investigation of CH and its consequences.

A limitation of this study was the lack of follow-up of individuals to confirm TSH screening values. We also performed ABR analysis at supra-threshold levels, and a deterioration in the auditory evoked response could be more evident at low levels (as it recruits fewer neurons). Furthermore, we did not evaluate ABR wave amplitudes, which might have shed further light on the differences in the central auditory system development and its consequences [27].

A larger sample could have resulted in a higher test power or allowed the detection of a smaller mean difference between waves and intervals; it also could have permitted a more accurate analysis of the correlation between wave I and TSH, as r values lower than 0.3 usually require a high sample size.

One of the strengths of this study was the rigorous selection of the sample population, which excluded individuals with factors that could have affected the maturation of the auditory system (risk indicators for hearing impairment, anemia, iron deficiency, latent iron deficiency, and significant use of alcohol or smoking during pregnancy). We also excluded newborns of mothers who had a diagnosed thyroid pathology and/or used levothyroxine during pregnancy. In addition, the perinatal conditions were homogeneous between the groups, reinforcing our results.

Other studies that sought to correlate hypothyroidism with ABR outside the neonatal period were contradictory; some showed increased wave latency and interpeak intervals, while others did not [9, 28]. In a mini review of TH and SNC, Prezioso et al. (2018) concluded that CH is demonstrably associated with changes in neurodevelopment [3]. On the other hand, other authors concluded in their study that the changes found in the ABR test of hypothyroid patients could be explained by the lower body temperature of these individuals, with no direct causal relationship with thyroid hormone levels [29]. These data are scarcer and more conflicting for subclinical hypothyroidism (SCH). Recently, it was shown that adult women with SCH had increased absolute latency of waves I, III, and V in ABR and increased interpeak latency of intervals III–V and I–V bilaterally, when compared to age- and sex-matched controls [30]. Paladugu et al. (2015) also showed an impairment of the cognitive function in individuals aged 12 to 45 years by analyzing evoked auditory potentials (P300), with increased latency in patients with SCH. The author concluded that there are few studies analyzing the cognitive function of hypothyroid patients through objective methods, such as the analysis of evoked potentials, and that more studies are needed to clarify this theme [31].

Therefore, although much is currently known about CH, and severe neurological sequelae have been prevented with the recommended treatment, many questions remain to be clarified. These issues include the ideal cut-off for TSH values in neonatal screening programs and the spectrum of structural and functional neurological changes found in subclinical and appropriately treated hypothyroid patients [7].

In conclusion, this study has contributed to the investigation of TSH relationship with auditory myelinization evaluated by ABR. It did not show a significant change in lower auditory pathway myelinization according to TSH in RN with TSH screening levels lower than 15μUI/mL Moreover, the study design and methodology used in the present study to analyze the relationship between TSH levels and CNS development, by evaluating lower auditory pathway myelinization, proved adequate and feasible in the neonatal period.

## Supporting information

S1 Fig Flowchart. Flowchart detailing exclusion criteria.
(PDF)

S1 File Multiple regression analysis. Multiple regression analysis with TSH as the dependent variable, and waves (I, III, and V) and intervals (I-III, III-V, and I–V) as the independent variables.
(PDF)
