# Supplementary figures and images for: Neonatal thyrotropin levels and auditory neural maturation in full-term newborns
Source: PLoS One. 2021 Jun 16;16(6):e0253229. doi: 10.1371/journal.pone.0253229 (PMC8208557; doi:10.1371/journal.pone.0253229)

**S1 Fig.** Flowchart detailing exclusion criteria.

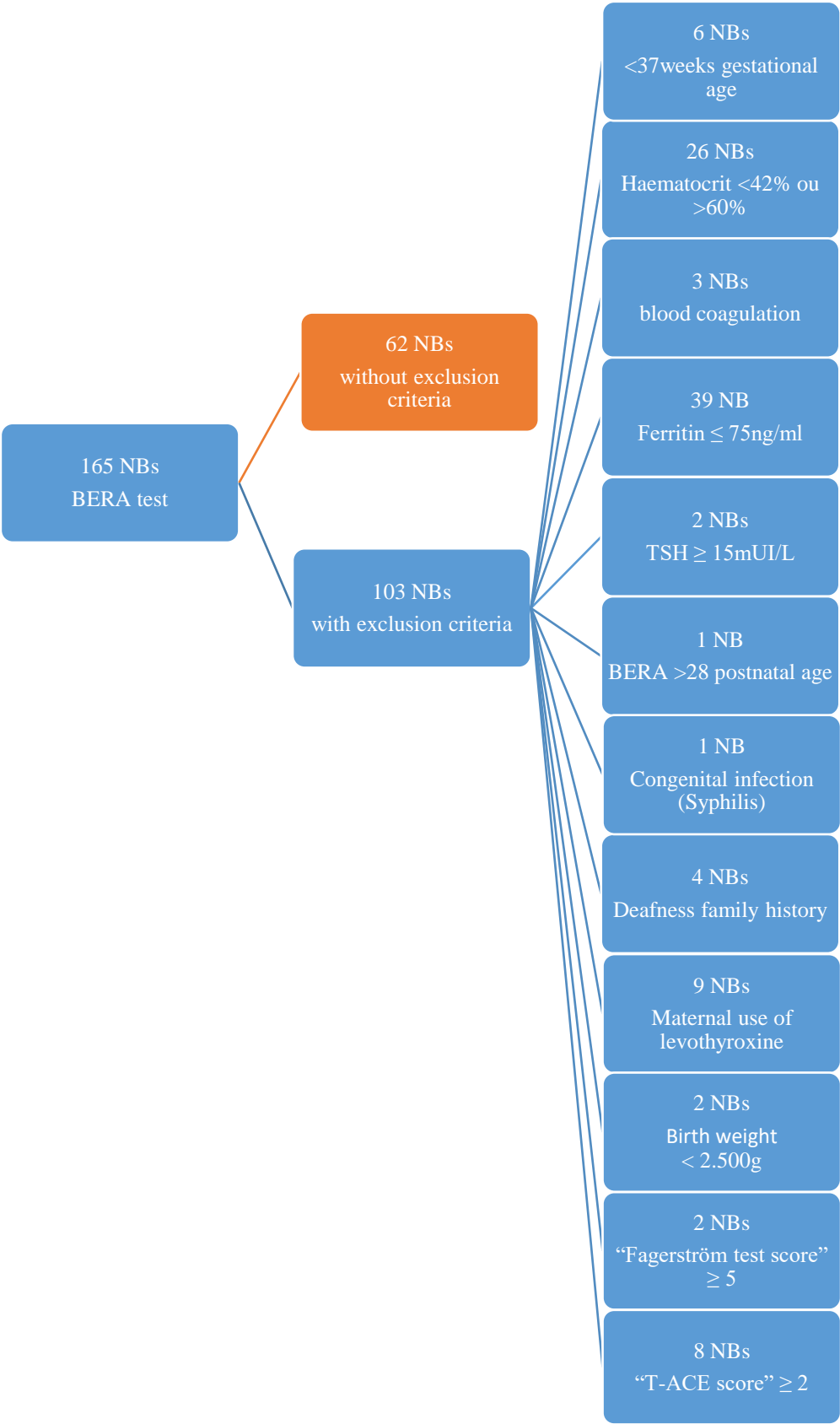

Supplement: S1 Fig — Flowchart detailing exclusion criteria. (PDF) [file pone.0253229.s001.pdf]
