# Supplementary material for: Neonatal thyrotropin levels and auditory neural maturation in full-term newborns
Source: PLoS One. 2021 Jun 16;16(6):e0253229. doi: 10.1371/journal.pone.0253229 (PMC8208557; doi:10.1371/journal.pone.0253229)
Supplement: S1 File — Multiple regression analysis with TSH as the dependent variable, and waves (I, III, and V) and intervals (I-III, III-V, and I–V) as the independent variables. (PDF) [file pone.0253229.s002.pdf]

```
REGRESSION
  /DESCRIPTIVES MEAN STDDEV CORR SIG N
  /MISSING LISTWISE
  /STATISTICS COEFF OUTS CI(95) R ANOVA COLLIN TOL ZPP
  /CRITERIA=PIN(.05) POUT(.10)
  /NOORIGIN
  /DEPENDENT TSH
  /METHOD=STEPWISE ONDAI ONDAIII ONDAV INTERI5 INTERI3 INTER35
  /SCATTERPLOT=(*ZRESID ,*ZPRED)
  /RESIDUALS DURBIN HISTOGRAM(ZRESID) NORMPROB(ZRESID)
  /CASEWISE PLOT(ZRESID) OUTLIERS(3).
```

Regression

Notes

Output Created		18-MAR-2021 09:39:01
Comments		
Input	Data	C:\Users\Carla_PC\Desktop\TSH.sav
	Active Dataset	DataSet1
	Filter	<none>
	Weight	<none>
	Split File	<none>
	N of Rows in Working Data File	62
Missing Value Handling	Definition of Missing	User-defined missing values are treated as missing.
	Cases Used	Statistics are based on cases with no missing values for any variable used.

Notes

Syntax		REGRESSION<br>/DESCRIPTIVES MEAN STDDEV CORR SIG N<br>/MISSING LISTWISE<br>/STATISTICS COEFF OUTS CI(95) R ANOVA COLLIN TOL ZPP<br>/CRITERIA=PIN(.05) POUT(.10)<br>/NOORIGIN<br>/DEPENDENT TSH<br>/METHOD=STEPWISE ONDAI ONDAIII ONDAV INTERI5 INTERI3 INTER35<br>/SCATTERPLOT=(*ZRESID ,*ZPRED)<br>/RESIDUALS DURBIN HISTOGRAM(ZRESID) NORMPROB(ZRESID)<br>/CASEWISE PLOT (ZRESID) OUTLIERS(3).
Resources	Processor Time	00:00:00.27
	Elapsed Time	00:00:00.27
	Memory Required	3860 bytes
	Additional Memory Required for Residual Plots	872 bytes

[DataSet1] C:\Users\Carla_PC\Desktop\TSH.sav

Descriptive Statistics

	Mean	Std. Deviation	N
TSH	5.1371	2.71144	62
ONDAI	1.7469	.10229	62
ONDAIII	4.3787	.12173	62
ONDAV	6.8824	.14466	62
INTERI5	2.4313	.10931	62
INTERI3	2.6082	.10032	62
INTER35	5.1031	.10759	62

Correlations

		TSH	ONDAI	ONDAIII	ONDAV	INTERI5	INTERI3
Pearson Correlation	TSH	1.000	-.267	-.165	-.118	-.211	.146
	ONDAI	-.267	1.000	.637	.689	.173	-.217
	ONDAIII	-.165	.637	1.000	.700	-.003	.553
	ONDAV	-.118	.689	.700	1.000	.442	.140
	INTERI5	-.211	.173	-.003	.442	1.000	-.231
	INTERI3	.146	-.217	.553	.140	-.231	1.000
	INTER35	.081	.069	.385	.715	.472	.398
Sig. (1-tailed)	TSH	.	.018	.100	.180	.050	.128
	ONDAI	.018	.	.000	.000	.089	.045
	ONDAIII	.100	.000	.	.000	.489	.000
	ONDAV	.180	.000	.000	.	.000	.139
	INTERI5	.050	.089	.489	.000	.	.035
	INTERI3	.128	.045	.000	.139	.035	.
	INTER35	.266	.298	.001	.000	.000	.001
N	TSH	62	62	62	62	62	62
	ONDAI	62	62	62	62	62	62
	ONDAIII	62	62	62	62	62	62
	ONDAV	62	62	62	62	62	62
	INTERI5	62	62	62	62	62	62
	INTERI3	62	62	62	62	62	62
	INTER35	62	62	62	62	62	62

Correlations

		INTER35
Pearson Correlation	TSH	.081
	ONDAI	.069
	ONDAIII	.385
	ONDAV	.715
	INTERI5	.472
	INTERI3	.398
	INTER35	1.000
Sig. (1-tailed)	TSH	.266
	ONDAI	.298
	ONDAIII	.001
	ONDAV	.000
	INTERI5	.000
	INTERI3	.001
	INTER35	.
N	TSH	62
	ONDAI	62
	ONDAIII	62
	ONDAV	62
	INTERI5	62
	INTERI3	62
	INTER35	62

Variables Entered/Removed[a]

Model	Variables Entered	Variables Removed	Method
1	ONDAI	.	Stepwise (Criteria: Probability-of-F-to-enter <= .050, Probability-of-F-to-remove >= .100).

a. Dependent Variable: TSH

Model Summary[b]

Model	R	R Square	Adjusted R Square	Std. Error of the Estimate	Durbin-Watson
1	.267[a]	.071	.056	2.63484	1.774

a. Predictors: (Constant), ONDAI

b. Dependent Variable: TSH

ANOVA[a]

Model		Sum of Squares	df	Mean Square	F	Sig.
1	Regression	31.921	1	31.921	4.598	.036[b]
	Residual	416.544	60	6.942		
	Total	448.465	61			

a. Dependent Variable: TSH

b. Predictors: (Constant), ONDAI

Coefficients[a]

Model		Unstandardized Coefficients		Standardized Coefficients	t	Sig.
		B	Std. Error	Beta		
1	(Constant)	17.491	5.771		3.031	.004
	ONDAI	-7.072	3.298	-.267	-2.144	.036

Coefficients[a]

Model		95.0% Confidence Interval for B		Correlations			Collinearity Statistics
		Lower Bound	Upper Bound	Zero-order	Partial	Part	Tolerance
1	(Constant)	5.947	29.034				
	ONDAI	-13.668	-.475	-.267	-.267	-.267	1.000

Coefficients[a]

Model		Collinearity ...
		VIF
1	(Constant)	
	ONDAI	1.000

a. Dependent Variable: TSH

Excluded Variables[a]

Model		Beta In	t	Sig.	Partial Correlation	Collinearity Statistics	
						Tolerance	VIF
1	ONDAIII	.008[b]	.051	.959	.007	.594	1.683
	ONDAV	.125[b]	.724	.472	.094	.526	1.903
	INTERI5	-.170[b]	-1.353	.181	-.173	.970	1.031
	INTERI3	.093[b]	.724	.472	.094	.953	1.050
	INTER35	.100[b]	.798	.428	.103	.995	1.005

Excluded Variables[a]

Model		Collinearity ... Minimum Tolerance
1	ONDAIII	.594
	ONDAV	.526
	INTERI5	.970
	INTERI3	.953
	INTER35	.995

a. Dependent Variable: TSH

b. Predictors in the Model: (Constant), ONDAI

Collinearity Diagnostics[a]

Model	Dimension	Eigenvalue	Condition Index	Variance Proportions	
				(Constant)	ONDAI
1	1	1.998	1.000	.00	.00
	2	.002	34.463	1.00	1.00

a. Dependent Variable: TSH

Residuals Statistics[a]

	Minimum	Maximum	Mean	Std. Deviation	N
Predicted Value	3.4182	6.6712	5.1371	.72339	62
Residual	-3.78614	7.79672	.00000	2.61316	62
Std. Predicted Value	-2.376	2.121	.000	1.000	62
Std. Residual	-1.437	2.959	.000	.992	62

a. Dependent Variable: TSH

Charts

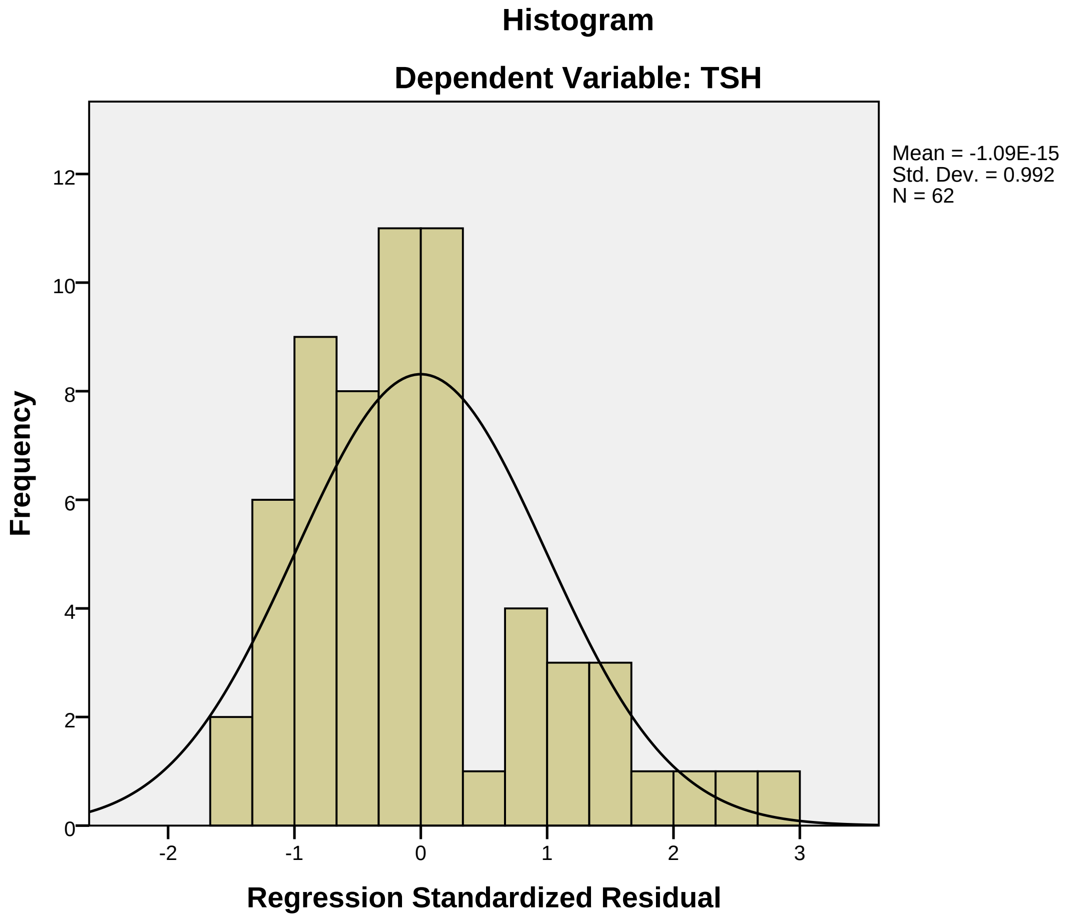
Histogram
Dependent Variable: TSH
Mean = -1.09E-15
Std. Dev. = 0.992
N = 62
Frequency
0
2
4
6
8
10
12
-2
-1
0
1
2
3
Regression Standardized Residual

Normal P-P Plot of Regression Standardized Residual

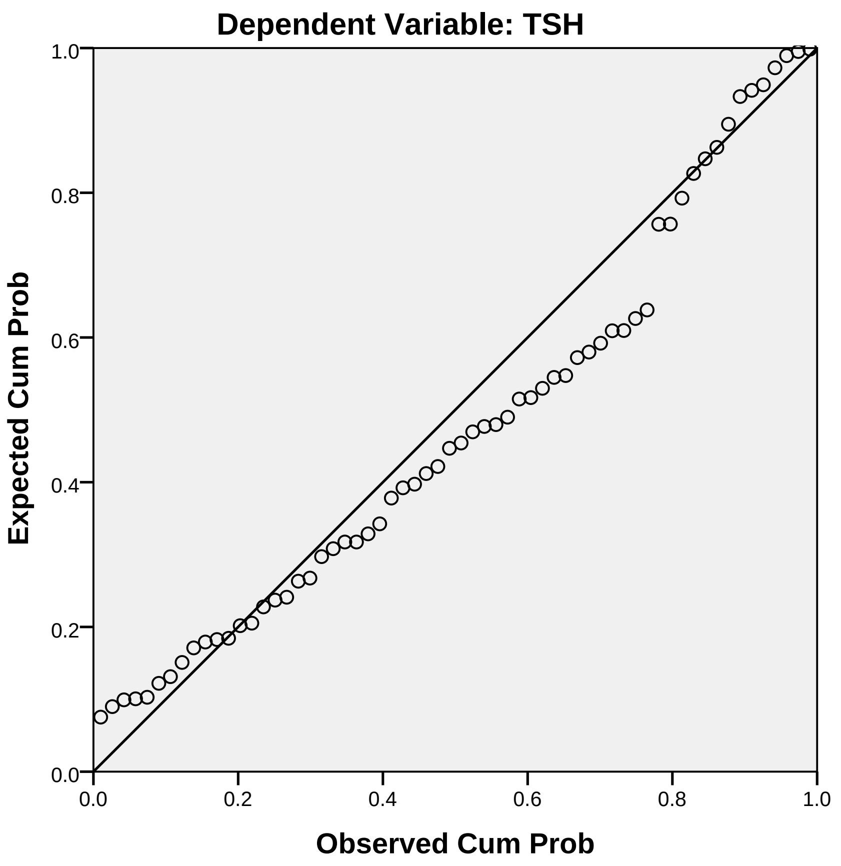

Scatterplot

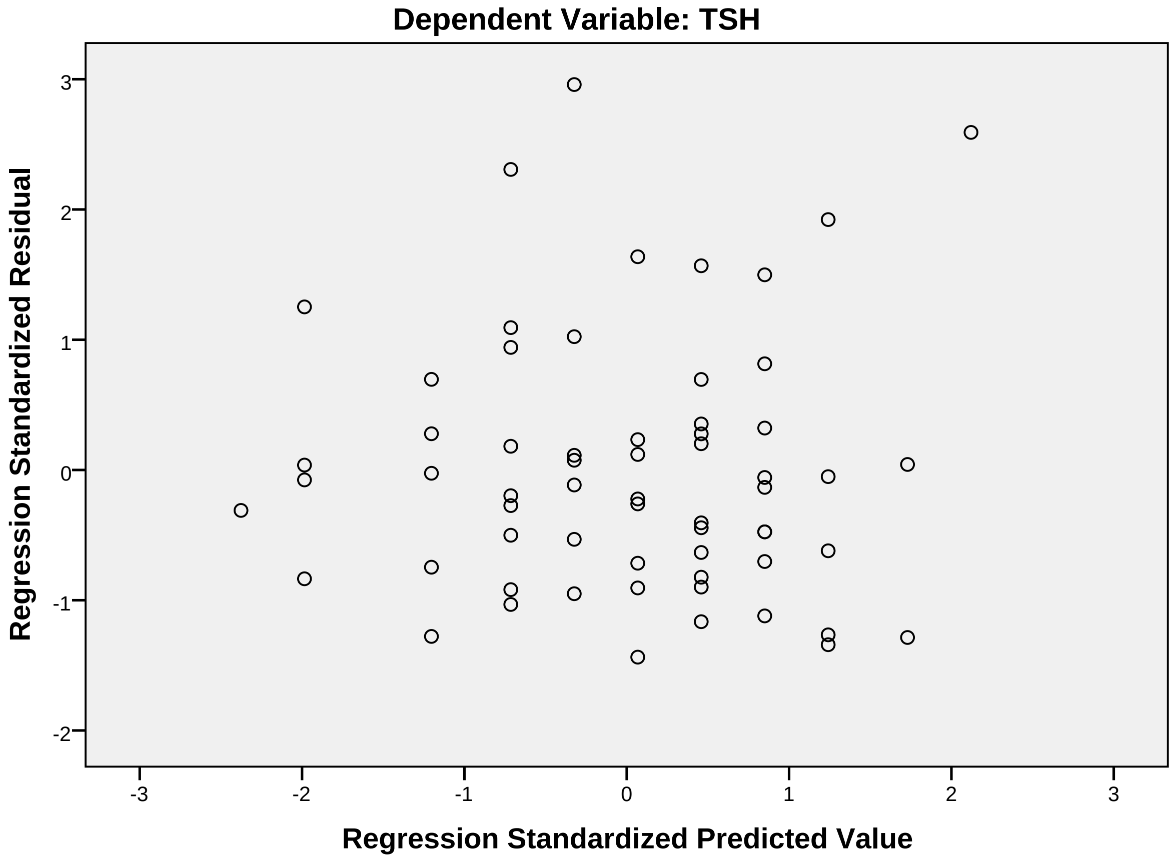


```
DATASET COPY  DataSet2 WINDOW=FRONT.
```
